# Effect of a Virtual Reality Exercise on Patients Undergoing Haemodialysis: A Randomised Controlled Clinical Trial Research Protocol

**DOI:** 10.3390/ijerph20054116

**Published:** 2023-02-25

**Authors:** Erika Meléndez-Oliva, Eleuterio A. Sánchez-Romero, Eva Segura-Ortí, José-Antonio Gil-Gómez, Xabier A. Soto-Goñi, Emilio J. Poveda-Pagán

**Affiliations:** 1 Center for Translational Research in Physiotherapy, Miguel Hernández University, San Juan, 03550 Alicante, Spain; 2 Department of Physiotherapy, Faculty of Sport Sciences, European University of Madrid, Villaviciosa de Odón, 28670 Madrid, Spain; 3 Physiotherapy and Orofacial Pain Working Group, Sociedad Española de Disfunción Craneomandibular y Dolor Orofacial (SEDCYDO), 28009 Madrid, Spain; 4 Musculoskeletal Pain and Motor Control Research Group, Faculty of Sport Sciences, European University of Madrid, 28670 Madrid, Spain; 5 Department of Physiotherapy, Faculty of Health Sciences, Universidad Europea de Canarias, 38300 Santa Cruz de Tenerife, Spain; 6 Musculoskeletal Pain and Motor Control Research Group, Faculty of Health Sciences, Universidad Europea de Canarias, 38300 Santa Cruz de Tenerife, Spain; 7 Department of Physiotherapy, Universidad Cardenal Herrera-CEU, CEU Universities, 46115 Valencia, Spain; 8 Instituto Universitario de Automática e Informática Industrial, Universitat Politècnica de València, 46022 Valencia, Spain; 9 Department of Psychobiology, Complutense University of Madrid, 28040 Madrid, Spain

**Keywords:** end-stage kidney disease, renal dialysis, inflammation, physical fitness, psychological wellness, physical activity, virtual reality

## Abstract

High levels of inflammatory markers have been associated with a greater deterioration of renal function and cardiovascular morbidity and mortality. For its part, physical exercise has been shown to be beneficial in improving the functional, psychological, and inflammatory states of patients with chronic kidney failure (CKF) undergoing haemodialysis (HD) treatment, improving their health-related quality of life. In recent years, virtual reality (VR) has been studied and described as an effective and safe tool that improves patients’ adherence to exercise programs. For these reasons, we propose to analyse the effect of VR exercise on the functional, psychological, and inflammatory states of patients on HD, as well as their levels of adherence to exercise, and compare them with static pedalling exercises. We will randomise 80 patients with CKF into two blind groups: an experimental group, which will carry out an intradialytic exercise program with non-immersive VR (n = 40), and a control group, which will exercise with a static pedal (n = 40). Functional capacity, inflammatory and phycological status, and exercise adherence will be analysed. Higher levels of adherence to exercise are expected in the VR group, which will have greater effects on the patients’ functional capacity and psychological and inflammatory status.

## 1. Introduction

Chronic kidney disease (CKD) represents one of the main public health problems [1]. It affects one in seven adults in Spain, being more prevalent in men (23.1% in men vs. 7.3% in women), in older populations (4.8% in subjects aged 18–44, 17.4% in subjects aged 45–64, and 37.3% in subjects ≥ 65 years), and in subjects with cardiovascular disease (39.8% in subjects with cardiovascular disease vs. 14.6% in subjects without cardiovascular disease) [2].

Globally, about 500 million adults suffer from CKD [3]. Although only 1% of these patients require renal replacement therapy (RRT) through dialysis or kidney transplantation, this condition leads to a marked reduction in life expectancy. Moreover, it constitutes one of the most expensive treatments for chronic diseases, consuming 5% of the budget of the healthcare system [4].

Patients with CKD present persistent low-grade inflammation, which is recognised as an important component in the pathophysiology of CKD and is responsible, in part, for cardiovascular mortality and general mortality, in addition to contributing to the development of protein-energy wastage [5].

There is an inversely proportional relationship between the glomerular filtration rate (GFR) and plasma levels of inflammatory markers caused by the presence of oxidative stress, endothelial dysfunction, and vascular calcification [6], together with additional factors such as periodontitis, accumulation of circulating cytokines, non-biocompatible haemodialysis membranes, excess volume, and subclinical infections of vascular access routes [7].

Different biomarkers of inflammation have been identified with predictive value in CKD/chronic kidney failure (CKF). In a large multicentre international database of HD patients, C-reactive protein (CRP) predicted mortality with an accuracy comparable to that of albumin and superior to ferritin and white blood cell (WBC) count [8]. However, it appears that interleukin 6 (IL-6) is a better predictor of cardiovascular and all-cause mortality than CRP and other cytokines, such as tumour necrosis factor alpha (TNF-α), interleukin 1-beta (IL-1β), and interleukin 18 (IL-18) [9].

On the other hand, adhesion molecules also play an important role in inflammatory processes. These are proteins located on the cell surface and are involved in the attachment of leukocytes, endothelial cells, and other extracellular matrices. The main groups of adhesion molecules are the selectins, the integrins, the immunoglobulin superfamily, and the cadherins. In CKD, the most widely studied adhesion molecules associated with inflammation, cardiovascular risk, and mortality are intercellular adhesion molecules-1 (ICAM-1) and vascular adhesion molecules-1 (VCAM-1). Increased levels of these markers predict overall mortality and cardiovascular mortality in patients with CKD not undergoing HD and in patients undergoing HD [10].

The existence of a relationship between endothelial-dependent vasodilation and the markers of inflammation in uremic patients has also been demonstrated, suggesting a pathogenic role of inflammation in the endothelial dysfunction associated with uraemia [11].

Given that cardiovascular disease (CVD) and infection are the main causes of morbidity and mortality in patients with CKD [12,13], the immune dysfunction that accompanies CKD represents a primary target for improving the results of therapeutic interventions.

Physical exercise has been widely studied in recent years, having demonstrated its potential as a therapeutic tool in patients with CKF on HD [14,15]. Regular physical exercise of moderate intensity has a beneficial effect, improving immune function and exerting anti-inflammatory effects [16,17,18]. This modality of exercise has been associated with modulating cytokines, lowering levels of pro-inflammatory cytokines, and increasing levels of anti-inflammatory cytokines [19], contributing, in part, to reducing the risk of infections and CVD [16,17].

The chronic inflammatory state and the immune imbalance that patients with CKF present leads to a loss of strength and muscle mass, impairing the functional capacity of these patients. Physical exercise can modify the inflammatory response through the mechanical stress that it produces on the muscle fibre, improving the processes of adaptation, remodelling, and causing the structural and functional repair of the skeletal musculature [20,21,22]. In addition, physical activity can produce a transient increase in endogenous glucocorticoids, leading to a selective decrease in circulating CD16+ monocytes [23]. On the other hand, it has been shown that exercise regulates Toll-like receptors (TLRs) dependent on inflammation, decreasing their levels, which reduces the cellular expression of TLR4, attenuating the inflammatory response induced by lipopolysaccharides [24].

Similarly, patients with CKF on HD have very high levels of sedentary lifestyle, which exacerbates their chronic inflammatory state and increases the loss of muscle mass and strength. This situation, associated with their high cardiovascular (CV) morbidity and mortality, places these patients in a vicious cycle where exercise can bring them great benefits [25].

Physical exercise has been widely shown to improve the functional capacity of patients with CKF, either through intradialytic exercise [26] or home exercise programs [27].

Psychological problems are also very frequent in these patients, presenting a prevalence of depression of 27%, which is associated with impaired quality of life and increased mortality. Besides, there is evidence of an association of higher pro-inflammatory and lower anti-inflammatory cytokine levels and depressive symptoms in patients with CKD/CKF [28]. In this sense, exercise offers great benefits to improve both inflammatory levels and associated depressive symptoms [18,29].

In recent years, virtual reality (VR) has been recognised as a new and effective tool to promote physical activity and healthy behaviour, and its use in health promotion is increasing [30]. Recent research has shown that VR enhances the psychological benefits of exercise and increases the likelihood of greater long-term exercise adherence [31].

Recently, the integration of VR into traditional physical exercise and rehabilitation programs has aroused great interest in the fields of kinesiology and public health. Using VR, it is possible to intensify repetitive tasks and increase visual and auditory feedback, which makes it a novel and attractive therapeutic tool that can be combined with traditional physiotherapy, as it is safe and effective for the patients who use it [32].

Researchers have recently begun to investigate whether the beneficial effects that VR has been shown to have in exercise programs for other populations can be extrapolated to patients with CKF undergoing HD treatment. There are five clinical trials published to date [33,34,35,36,37], the results of which show that the application of VR exercise during HD improves physical function and reduces fatigue and depression in these patients.

In 2019, Segura-Orti and García-Testal conducted a review that reinforced these results, concluding that intradialytic exercise with VR is safe and improves physical function and health-related quality of life in patients on HD. They also concluded that this exercise can be performed safely during the last hours of the HD session [38].

Along the same lines, another review by Olumuyiwa et al. concludes that the use of VR during HD treatment significantly improves the levels of physical activity and fatigue in patients with CKF, as well as increases the levels of adherence and commitment to HD treatment, without the appearance of adverse effects [39].

Currently, only one study has analysed the effect of VR exercise on depression levels in patients on HD, whereas the effect of VR exercise on the inflammatory status of these patients has not been studied at all. It could be expected that the beneficial effects on inflammation are comparable to those obtained with conventional exercise [19], and their psychological state also improves. Therefore, we plan to carry out a study of these characteristics with the aim of determining the effectiveness of an intradialytic exercise program with VR on inflammatory markers, in addition to delving into its effect on functional capacity and psychological state. As a secondary objective, we intend to analyse the levels of adherence to the exercise program and compare the results obtained for all the variables to those of a group that performs intradialytic static pedal exercise.

## 2. Materials and Methods

### 2.1. Type of Study

**Figure 1:**
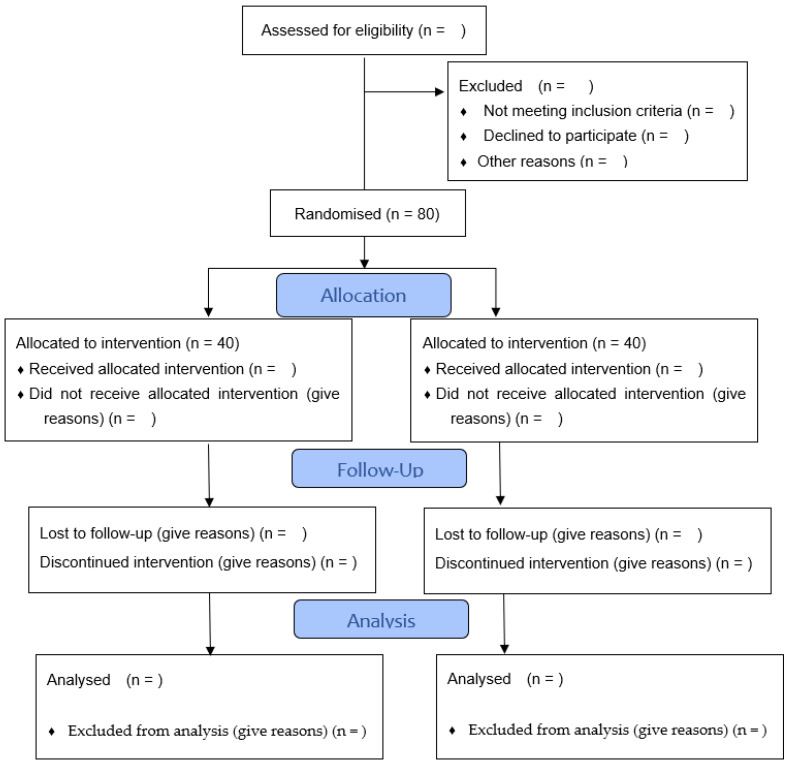
Flowchart of sample selection (obtained and modified from CONSORT 2010 Statement).

The study will be carried out prospectively between June 2023 and June 2024. It is an experimental, controlled, blind, and randomised study, whose methodology complies with the CONSORT standards for clinical trials. (CONSORT 2010 flow diagram, Figure 1). This protocol report follows the Standard Protocol Items: Recommendations for Interventional Studies (SPIRIT) 2013 checklist [40].

The study has passed the responsible research evaluation of Miguel Hernandez University of Alicante, with assigned code TFM.MMC.EJPP.EMO.220221, and later registered in ClinicalTrials.gov (NCT05606484) (accessed on 25 September 2022).

Before the study begins, the approval of the ethics committee of the Hospital General Universitario de Alicante Doctor Balmis will be requested.

### 2.2. Study Population

#### 2.2.1. Population Sample

The population sample will be selected from the Haemodialysis Unit of the General University Hospital of Alicante Doctor Balmis, which is visited by patients with terminal CRF, through convenience sampling. The following inclusion and exclusion criteria will be applied:

##### Inclusion Criteria [23,34]:

Patients with CKF in a stable clinical situation who have been on HD treatment for at least 3 months.

##### Exclusion Criteria [23,34]:

- Had a myocardial infarction in the 6 months before the start of the study;
- Suffered unstable angina at rest or during exercise;
- Have cerebrovascular diseases (such as transient ischemia or stroke);
- Present amputation of lower limbs above the knee without prothesis;
- Present a musculoskeletal or respiratory pathology likely to worsen with exercise;
- Present an inability to carry out functional assessment tests;
- Present visual or cognitive impairments that affect the ability to use the VR equipment or understand the VR exercise.

#### 2.2.2. Randomisation of the Sample

To avoid potential biases that could convolute the results of the program based on sex or age, the groups will be homogeneous in these characteristics. For this, randomisation will be carried out by blocks of sex and age (www.randomization.com), whose randomisation sequence will be hidden (OSA).

#### 2.2.3. Description of the Intervention

An intradialytic exercise program with non-immersive VR will be carried out for 12 weeks with the aim of improving the functional capacity of patients with CKF, as well as their psychological state (decreased anxiety and depression) and inflammatory state (decreased inflammatory markers IL-6, TNF-α, CRP, ICAM-1, and VCAM-1). For its execution, a sample of 80 subjects from the Haemodialysis Unit of the Balmis Hospital in Alicante will be selected, and the sample will be randomized into two groups (intervention group and control group). The intervention group (n = 40) will perform intradialytic exercise with non-immersive VR. The control group (n = 40) will perform intradialytic exercise with a static pedal.

As the health benefits of exercise in patients with CKF have been widely demonstrated [17,18,19], it would not be ethical to deprive one of the groups of exercising. Therefore, it has been decided that the control group will perform an intradialytic exercise with a static pedal, which will exercise the same muscle groups as in the intervention group with VR.

### 2.3. Exercise Program

The nursing staff from the Haemodialysis Unit and several physiotherapists from the Rehabilitation Unit of the Doctor Balmis General Hospital in Alicante will be instructed at the beginning of the study, so they become thoroughly familiar with the characteristics of both exercise programs (the VR exercise program and pedal exercise program). Later, they will be the ones who supervise and direct the exercise programs during the study.

During the two weeks before the start of the intervention, a test period will be carried out as a first contact, during which the nursing staff will implement the exercise programs to familiarise themselves with the material that the patients will use and to resolve possible doubts that may arise during the execution of each exercise. This trial period will be supervised by the specialist physiotherapist in charge of training the hospital’s nursing staff and physiotherapists.

Additionally, with the aim of improving the levels of adherence to the exercise program by the patients, a psychologist specialised in managing barriers during exercise in special populations will be assigned. They will give a talk to the staff in charge of supervising the intervention about the development of new strategies that help maximise levels of adherence to the program.

The exercise program will last for 12 weeks, with a frequency of three weekly exercise sessions of 30 min each. Exercise will take place during the first two hours of HD treatment [27] and patients will be instructed to exercise as independently as possible. Heart rate and blood pressure will be recorded before and after each session.

#### Description of the Non-Immersive VR Exercise Program

Patients will begin with a 5 min warm-up, followed by a 30 min exercise session with VR, with a perceived exertion between 12 and 15 (somewhat hard/hard) on the Borg rating of perceived exertion (RPE). The HD treatment can be carried out simultaneously as the exercises focus on the work of the lower limbs. The muscles that will be mainly exercised are the quadriceps femoris, adductors, abductors, triceps surae, abdominal muscles, and iliac psoas.

The VR used in the study will consist of an adapted version of ACT (*Treasure Hunt*). This is a non-immersive VR system with a playful design that makes it possible to liven up the HD sessions for patients who come for treatment. It consists of a game in which the patient must move their lower limbs, simulating a treasure hunt, where they must hunt some objects and avoid contact with others. The exercise allows the patient to alternate legs when they are tired. The difficulty of the game may be adjusted according to the characteristics of the participants by the means of hardware in a computer or television (usually present in the Haemodialysis Unit) and a Microsoft Kinect^®^ movement tracking system.

The VR session will be defined by the therapists at the beginning of the session through a management tool, where they will schedule rest periods and their duration, as well as the difficulty of the session based on the patient´s performance. Before the start of the first session, the participants will be instructed about the use of the VR system and a test session will be held to confirm that they have understood the execution of the program.

**Figure 2:**
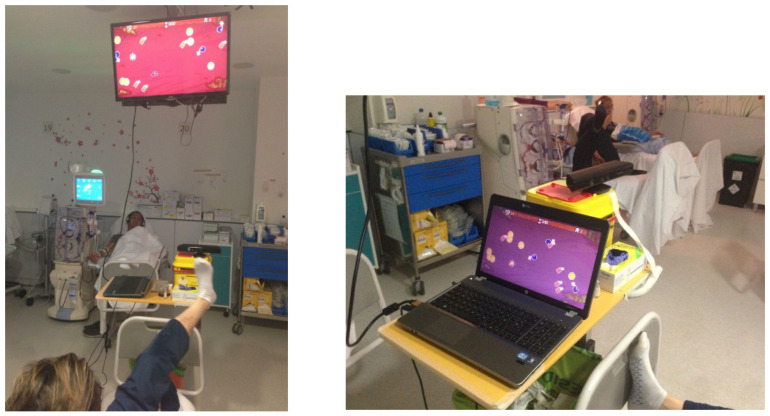
Intradialytic exercise with non-immersive VR (reproduced with permission from Segura-Ortí, E. et al., 2019) [34].

Once the exercise session is over, the patient will perform a gentle stretching session for 5 min. The intradialytic exercise with non-immersive VR to be performed can be seen in Figure 2.

**Figure 3:**
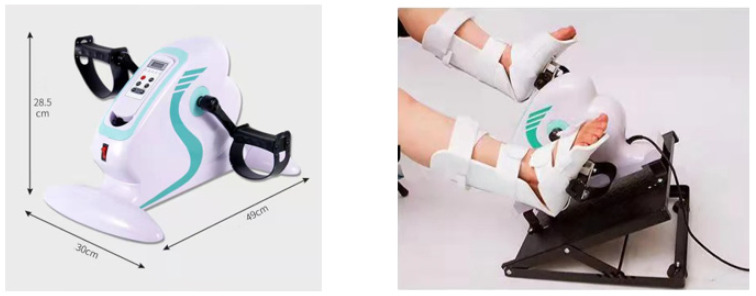
Static pedal for intradialytic exercise in the control group (obtained from Healthy Care Monitor Store).

Control group: The participants in this group will exercise with an electric rehabilitation training pedal attached to the bed or HD chair (Figure 3).

The exercise will be performed simultaneously with the non-immersive VR exercise group during the first two hours of HD treatment. As in the other group, three weekly sessions of 30 min will be carried out at an intensity at which the patient perceives an effort between 12 and 15 (somewhat hard/hard) on the RPE scale.

The muscles exercised with this program are the same as those exercised with the VR exercise (quadriceps femoris, triceps surae, adductors, abductors, iliopsoas, and abdominals).

Before the beginning of the exercise, 5 min of warm-up with light stretching will be performed and, at the end of the 30 min, it will be repeated. The static pedal to be used in the study are shown in Figure 3.

Both groups will carry out their exercise programs in the HD room of the hospital, which has separate departments where the patients of one group will not be able to see the exercise performed by the other group.

### 2.4. Functional Assessment of Patients

Functional tests will be carried out on three interspersed days one week before the start of the exercise program, and the tests will coincide with the days that patients received their HD treatment. Patients will be scheduled half an hour before their treatment to perform the corresponding functional tests.

All tests will be repeated when the exercise program is finished at 12 weeks.

#### 2.4.1. First Day of Functional Tests

##### Short Physical Performance Battery (SPPB) Test

**Figure 4:**
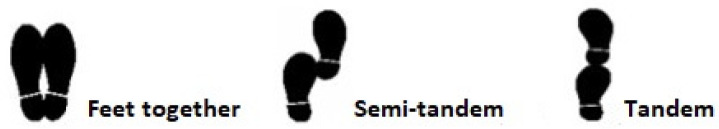
Balance tests (reproduced with permission from Erika Meléndez-Oliva 2020) [43].

Numerous longitudinal epidemiological studies have demonstrated the ability of SPPB to predict dependency, institutionalisation, hospitalisation, and mortality. The scores range from 1 to 12 points and consist of the following tests [41,42]:

- Three balance tests: Subjects stand with feet together, in semi-tandem, and in tandem, showing whether they can maintain that position for 10 s (Figure 4; score from 0 to 4).

**Figure 5:**
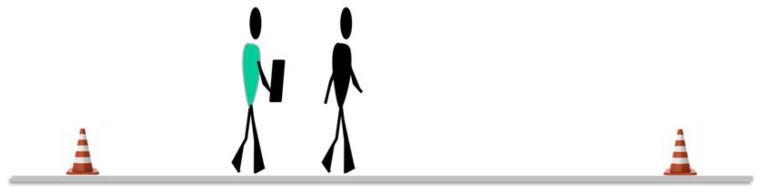
Walking Test at Normal Speed Over 4 Metres (reproduced with permission from Erika Meléndez-Oliva 2020) [43].

A Walking Test at Normal Speed Over 4 Metres: Two attempts are timed and the shortest of them is recorded. The test is scored from 0 to 4 based on the seconds taken (Figure 5).

- STS-5 Test: The test consists of getting up and sitting down five times on a chair without arms placed against the wall. The time taken until the patient is standing after the fifth repetition is measured, and a score from 0 to 4 is assigned. The test must be performed with arms crossed.

**Figure 6:**
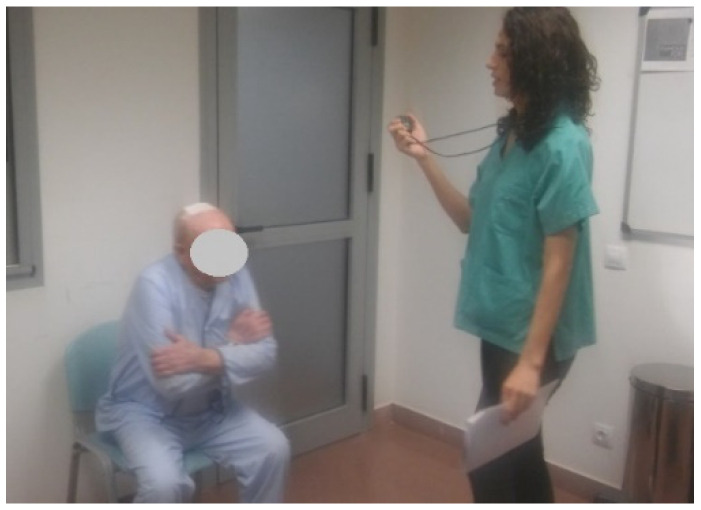
STS-5 Test (reproduced with permission from Erika Meléndez-Oliva 2020) [43].

The performance of the STS-5 Test can be seen in Figure 6.

- One Leg Balance Test: The test is assessed by asking the subjects to raise one leg (the one that gives them the most security), flex it, leaving the other supported, and holding it for as long as possible. The length of time is recorded at the end of the test. The subject can move the arms and flex the knee if necessary to maintain balance. The test ends when the subject uses the arms to support himself or the elevated leg or when 45 s has elapsed. The test is repeated three times and the best time is recorded [44].

**Figure 7:**
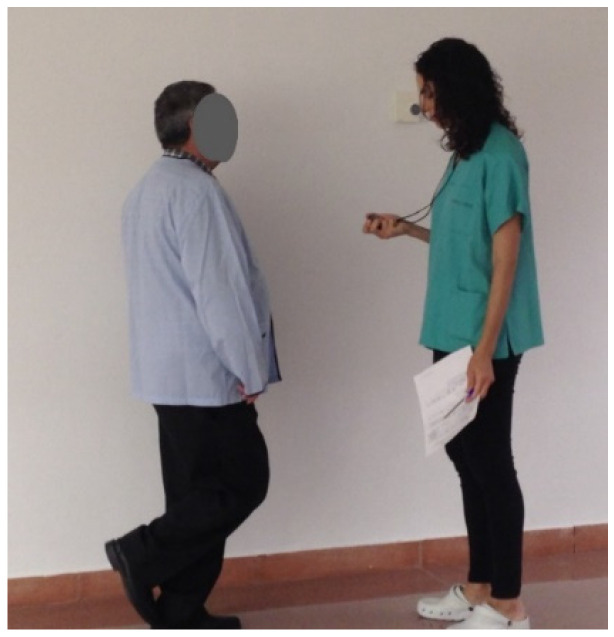
One Leg Balance Test (reproduced with permission from Erika Meléndez-Oliva 2020) [43].

The performance of the One Leg Balance Test can be seen in Figure 7.

**Figure 8:**
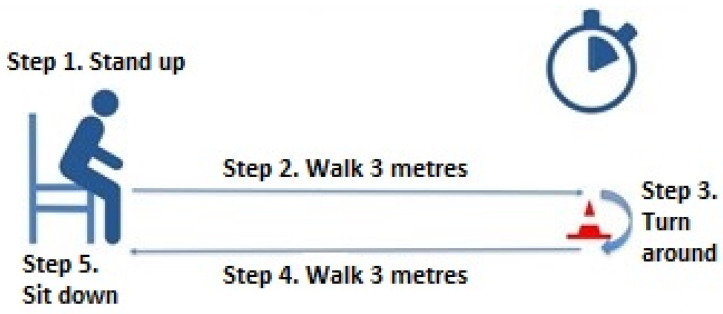
Timed Up and Go Test (reproduced with permission from Erika Meléndez-Oliva 2020) [43].

- Timed Up and Go Test: From a standard chair with armrests, the patient must get up, walk 3 metres, and return to the starting position. Time and effort are recorded on the RPE scale [42,45]. (Figure 8).

#### 2.4.2. Second Day of Functional Tests

- STS-10 Test [46]: The test measures the number of seconds it takes for the patient to get up and sit down 10 consecutive times in flat shoes. The test is performed on a chair without armrests, approximately 44.5 cm high, 38 cm deep, and leaning against the wall to minimise the risk of falling during the test [47]. The patient must remain seated for a few minutes before carrying out the test, allowing time between this and the previous test. The test must be carried out with the arms crossed over the chest. After the completion of the test, the time needed and the effort perceived with the RPE scale are recorded.
- STS-60 Test [48]: The patient must perform as many repetitions as possible for 60 s. After that time, the repetitions and the effort perceived on the RPE scale are recorded. The clinically important change for the STS-10 is 8.4 s, and that for the STS-60 is 4 repetitions [49].

**Figure 9:**
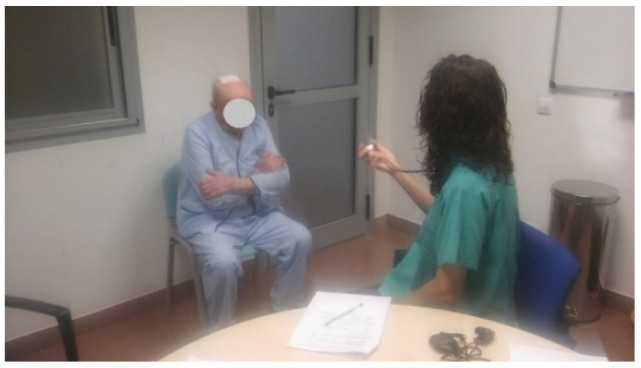
STS-10 and STS 60 Tests (reproduced with permission from Erika Meléndez-Oliva 2020) [43].

The performance of the STS-10 Test and the STS-60 Test can be seen in Figure 9.

#### 2.4.3. Third Day of Functional Tests

##### Six-Minute Walk Test [50,51]

**Figure 10:**
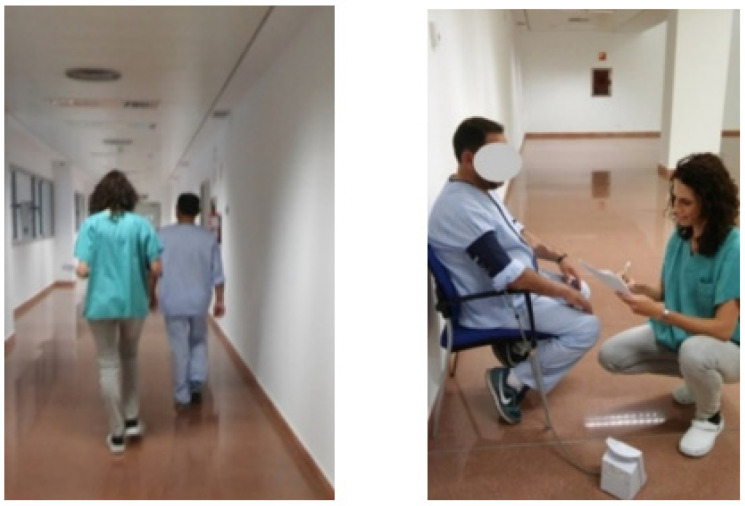
Six-Minute Walk Test (reproduced with permission from Erika Meléndez-Oliva 2020) [43].

The patient is informed in advance that they should wear comfortable shoes and that the test will be carried out in the corridor of the Haemodialysis Unit of the Alicante Hospital. A heart rate monitor continuously tracks the heart rate. Before performing the test and after resting for five minutes, baseline heart rate and blood pressure are recorded with an electronic blood pressure monitor. Then, the patient covers the pre-established distance as many times as possible in six minutes according to the existing space and markings on the ground (every two metres) and turning without stopping. The instruction that should be given to the patient is ‘walk as far as possible for six minutes’. For those patients who use walking aids in their daily life, they are allowed to carry out the test with them or the assistance of another person, and to stop and restart the march if they need to rest (without stopping the stopwatch). Immediately after the test, the heart rate, blood pressure, and distance travelled are recorded, and the patient is asked to describe the degree of test difficulty on the RPE scale. The clinically important change in this test is equivalent to 66.3 metres [51]. The performance of the Six-Minute Walk Test can be seen in Figure 10.

### 2.5. Plasma Determinations

#### Inflammatory Parameters

The inflammatory markers determined will be IL-6, TNF-α, CRP, ICAM-1, and VCAM-1. All inflammatory markers will be analysed using the enzyme-linked immunosorbent assay (ELISA) technique. The ELISA technique is based on the use of antigens and antibodies labelled with an enzyme, so that the resulting conjugates have both immunological and enzymatic activity. As one of the components (antigen or antibody) is labelled with an enzyme and insolubilized on a support (immunosorbent), the antigen–antibody reaction will be immobilised; therefore, it will be readily revealed by the addition of a specific substrate that, when the enzyme acts, will produce a colour observable by the naked eye or quantifiable by a spectrophotometer or a colorimeter [52].

### 2.6. Psychological Assessment

#### 2.6.1. Multidimensional Questionnaire of Adaptation to the Disease for Renal Patients on Dialysis (CAMAE-RD) [53]

The CAMAE-RD consists of Phase A and Phase B. Phase A, which includes 12 items, collects all of the information from the clinical history of the patient. This phase includes two areas: Data Doctors and Previous Psychopathological History. Phase B, which includes 51 items, is made up of nine areas that form the evaluation of the patient: Sociodemographic Data (six items), Information (nine items), Medication and Diet (three items), Social Support (four items), State of Encouragement (two items), Personal Resources, Coping and Resilience (eight items), Learning and Perceived Changes (five items), Spirituality (two items), and Care Planning (two items). There are four types of questions depending on the type of response: those with a closed character (dichotomous responses, nominal categorical, and 10-point Likert-type scales) and those with an open character (qualitative responses). The increase in the score on the scale with a statistically significant change (*p* < 0.05) between the pre-intervention and post-intervention value will be considered positive.

#### 2.6.2. State-Trait Anxiety Inventory (STAI) [54]

STAI is a survey composed of 20 items that assess state anxiety, STAI-E (transient emotional state), and another 20 items that assess trait anxiety, STAI-R (anxious, relatively stable propensity of the participant, in general).

#### 2.6.3. Beck Depression Inventory II (BDI-II) [55]

BDI-II is a tool that assesses the symptoms of depression. The questionnaire consists of 21 questions, providing a range of score between 0 and 63. The questionnaire proposes the following cut-off scores and corresponding degrees of depression: 0–13 indicates minimal depression; 14–19, mild depression; 20–28, moderate depression; and 29–63, severe depression.

### 2.7. Exercise Adherence

Adherence to exercise will be measured by counting the number of exercise sessions in which the patient participates. High adherence will be considered when a patient exceeds 75% of the scheduled sessions (27 of 36 sessions).

### 2.8. Statistical Analysis

The sample size of the study was calculated using the statistical program G-Power (analysis of variance (ANOVA): repeated measures, within/between interaction), assuming a type I error or α of 0.05 (95% confidence interval), a statistical power of 0.80 (type II or β error), and an effect size of 0.25 (mean), requiring the inclusion of 24 patients to ensure the external validity of the study. Considering that there may be losses during the study follow-up, 80 patients will be included: 40 in the control group and 40 in the intervention group.

Intention-to-treat statistical analysis will be performed using the statistical program SPSS 23.0 for Windows (SPSS Inc, Chicago, IL, USA).

The normal distribution of the groups will be verified through the chi-square and Mann–Whitney U tests for correct randomisation.

A mixed two-way ANOVA test will be used to analyse the effect of the VR exercise on functional variables and inflammatory variables in each study group and to analyse the differences between the two groups.

Data will be presented as mean ± standard deviation, setting statistical significance at *p* < 0.05.

## 3. Expected Results

Both exercise groups (with non-immersive VR and with a static pedal) are expected to see a decrease in their levels of inflammatory markers and improvement in their functional capacity and psychological status. However, higher levels of adherence are expected in the VR group, which will have greater effects on the variables studied in the VR exercise group than in the static-pedal-exercise group.

## 4. Conclusions

VR can improve the level of adherence to exercise in patients on HD. This means that the patients perform a greater number of exercise sessions than with conventional exercise, giving rise to a greater effect of the VR exercise on the inflammatory state, functional capacity, and psychological state of the patients; thus, this enhances their health-related quality of life and improves their cardiovascular morbidity and mortality.

In addition to these benefits, VR can also liven up the HD sessions for patients, making CKF more bearable for patients who undergo 4 h treatments three times a week.

## Data Availability

The data presented in this study are available upon request from the corresponding author.
